# Cystic Artery Stump Pseudoaneurysm Following Laparoscopic Cholecystectomy: A Case Report

**DOI:** 10.7759/cureus.64551

**Published:** 2024-07-15

**Authors:** Dalal Sibria, Mohamed Elawad, Loai J. A. Aker, Ali Barah, Omran Almokdad, Sushila B Ladumor, Maysa A Mohamed, Amal A Al Rashid, Yaman M Alahmad, Aalaa Kambal

**Affiliations:** 1 Radiology Department, Hamad Medical Corporation, Doha, QAT

**Keywords:** surgical ligation, embolization, hematochezia, pseudoaneurysm, cystic artery stump

## Abstract

Cystic artery stump pseudoaneurysm (CASP) is a potentially life-threatening condition that can be related to multiple etiologies, especially the iatrogenic factor owing to the increased number of hepatobiliary procedures. Most patients present with haemobilia. Here we report a successfully managed case of CASP that initially complained of right upper abdominal pain.

A 38-year-old patient developed bile duct injury after laparoscopic cholecystectomy (LC) which was identified by magnetic resonance cholangiopancreatography (MRCP). Later, she developed haemobilia due to CASP which was then treated by trans-arterial embolization (TAE).

CASP is a rare complication of post-LC, yet potentially life-threatening, with possible delayed complications occurring months to years after the surgery. Clinicians and radiologists should be aware of this important entity and its variable manifestations to facilitate early treatment.

## Introduction

A pseudoaneurysm is defined as an abnormal outpouching or dilatation of an artery that is bounded only by the outermost layer of the arterial wall (tunica adventitia), compared to a true aneurysm that is bound by all three layers of the arterial wall [1]. Pseudoaneurysm formation is a consequence of vascular injury caused by trauma, or more commonly iatrogenic causes, including hepatobiliary biopsies and surgeries [2]. We are reporting a case of pseudoaneurysm in the cystic artery (CA), which was diagnosed through computed tomography (CT) and angiographic evaluations and was treated with trans-arterial embolization (TAE).

This case report was previously posted to the Research Square preprint server on November 3, 2021.

## Case presentation

A 38-year-old female, with an unremarkable medical history, presented to the emergency department with chief complaints of right upper quadrant (RUQ) pain, nausea, and vomiting 11 days after laparoscopic cholecystectomy (LC). There was no evidence of fever, haematemesis, or melena. Bedside physical examination was positive for RUQ tenderness. Basic laboratory investigations were unremarkable except for cholestatic liver enzyme elevation.

**Figure 1:**
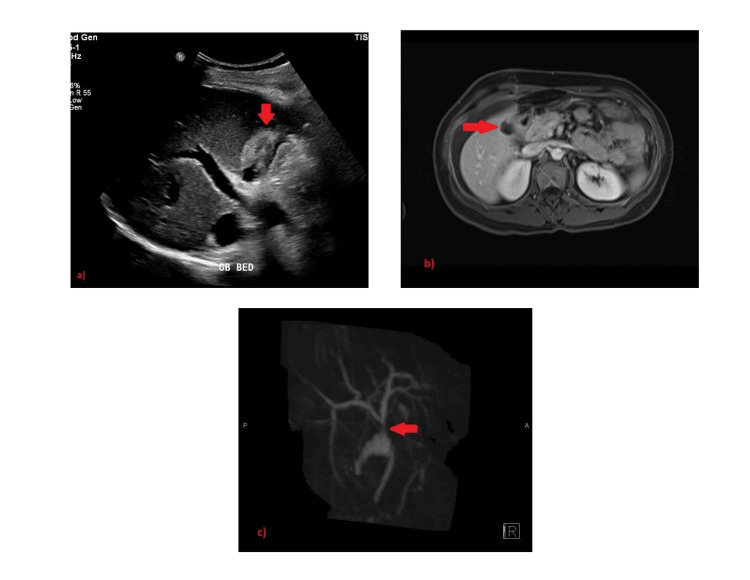
Selected images of ultrasound and magnetic resonance cholangiopancreatography showing gallbladder fossa collection. (a) Ultrasound abdomen shows a well-defined collection within the gallbladder fossa (arrow). (b) Axial T1 fat sat post-contrast axial image shows gallbladder fossa collection, with rim enhancement (arrow). (c) Coronal 3D reconstruction of magnetic resonance cholangiopancreatography image showing suspected communication between the collection’s upper part and adjacent hepatic duct confluence (arrow), concerning bile duct injury.

Ultrasound (US) scan of the abdomen showed a well-defined subhepatic collection within the gallbladder fossa and minimal abdominopelvic free fluid (Figure 1a). Subsequently, magnetic resonance cholangiopancreatography (MRCP) showed T2 heterogenous, and T1 hypointense gallbladder fossa collection, with rim enhancement (Figure 1b) and no internal restricted diffusion, with a suspected communication between the collection’s upper part and the adjacent hepatic duct confluence, concerning for bile duct injury (Figure 1c). The common hepatic duct and common bile duct were of normal caliber.

**Figure 2:**
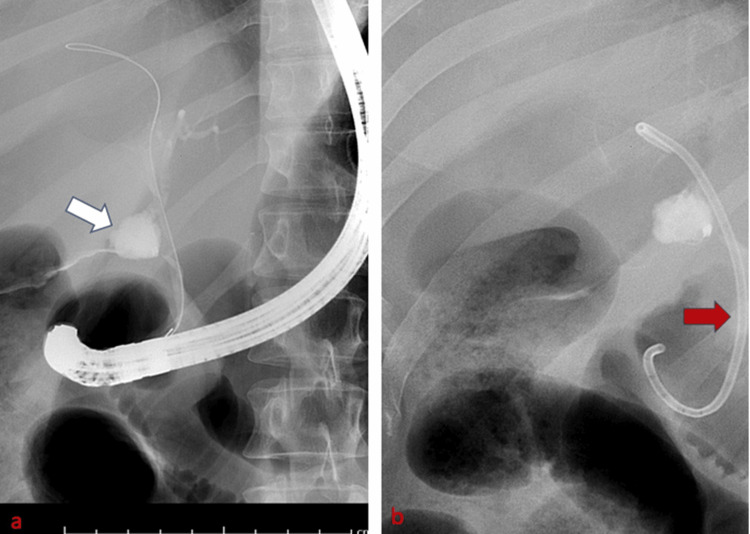
Selected images of endoscopic retrograde cholangiopancreatography Intraoperative radiographic images of endoscopic retrograde cholangiopancreatography (ERCP) demonstrating contrast leak (white arrow), after injecting contrast through the ampulla of Vater (a), concerning bile duct injury, followed by stent placement (red arrow) (b).

Subsequent endoscopic retrograde cholangiopancreatography (ERCP) confirmed bile duct injury and stricture (Figure 2a), and accordingly, a plastic stent was placed across the injury (Figure 2b).

CT-guided drainage of the abdominal collection was done by inserting an 8.3 French drainage catheter in the right subhepatic collection, followed by aspiration of 50 mL of orange/yellow bile with debris.

**Figure 3:**
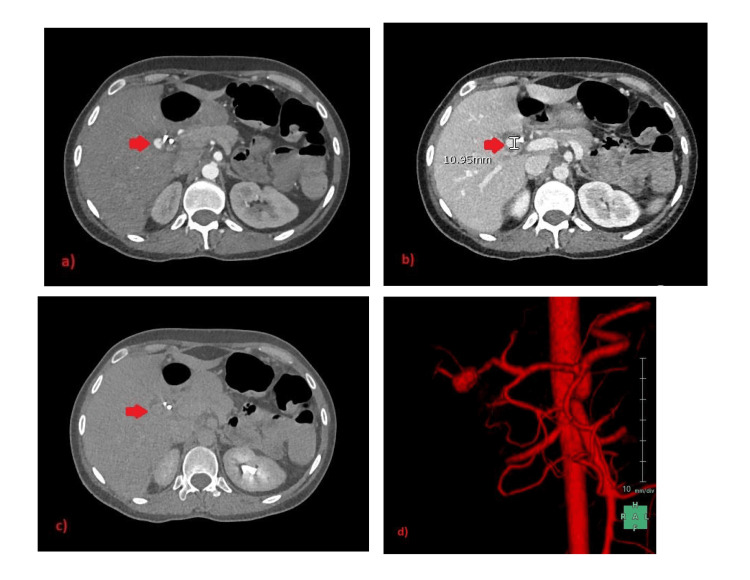
Selected images of computed tomography abdomen angiogram showing cystic artery stump pseudoaneurysm. Axial images of computed tomography (CT) abdominal angiogram study representing arterial (a), venous (b), and delayed acquisition phases (c), respectively, showing focal contrast-filled outpouching (arrows) at the porta hepatis along the lateral aspect of right hepatic artery closely related to cholecystectomy clip and biliary stent. The lesion appears iso-dense to the aorta on arterial phase image (a), and venous phase (b) and washing-out on a delayed scan (c), corresponding to a cystic artery stump pseudo aneurysm. (d) 3D reconstruction coronal view of the arterial phase on CT abdomen showing the origin of cystic artery stump pseudo aneurysm.

**Figure 4:**
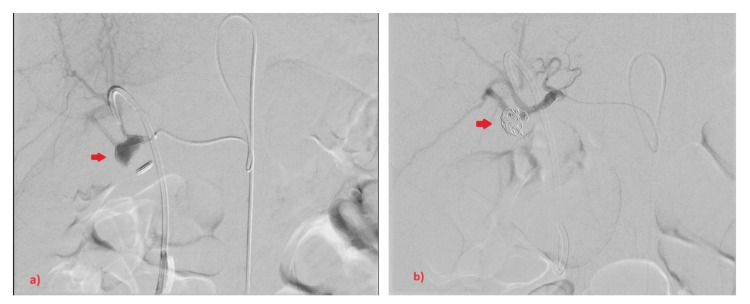
Selective common hepatic artery angiogram confirmed cystic artery stump pseudoaneurysm. Selective common hepatic artery angiogram confirmed cystic artery stump pseudoaneurysm (a), which was coiled by six micro coils (b).

A few days later, the patient developed itching, jaundice, fresh bleeding per rectum, melena, and an acute drop of hemoglobin of 3 mg/dL. Therefore, haemobilia was suspected. Subsequent abdominal CT angiogram showed a significant reduction in gallbladder fossa collection size, however, a suspicious small contrast-filled outpouching was seen at the porta hepatis, close to cholecystectomy clips, raising the possibility of a cystic artery stump pseudoaneurysm (CASP) (Figures 3a-3d). Hereafter, a selective common hepatic artery angiogram confirmed the presence of CASP, which was coiled by six micro coils (Figures 4a-4b). Thereafter, the patient recovered and was discharged home.

## Discussion

Hepatic artery or CA pseudoaneurysms are rare complications of LC, with CA involvement being reported much less frequently in the literature. It is hard to determine the incidence of the pseudoaneurysm, as it could be asymptomatic, thrombosed, or ruptured [2,3]. The reported time interval between the surgery and the onset of clinical symptoms is variable, with one month reported as the average time to presentation. However, a five-year delay in presentation has been described [2].

The exact mechanisms of hepatic or CA pseudoaneurysm after laparoscopic or open cholecystectomy are not completely identified up to date, however, they can be related to direct vascular injury (by applying surgical clips/thermal injury), adjacent gallbladder fossa bile collection that increases vessel wall fragility or as a consequence of post-surgical adhesions. Most of the cases presented with classical symptoms of haemobilia (gastrointestinal bleeding, upper abdominal pain, and jaundice) [4], but it is uncommon to present with symptoms of lower gastrointestinal bleeding [5].

Pseudoaneurysm is considered an acute emergency that requires immediate intervention. The diagnosis can be made by endoscopy, ERCP, CT, and conventional angiography, and managed primarily by TAE through occluding the sac or the feeding vessel with a variety of embolic agents, including coils, thrombin, or gel foam, before ideally embolizing the vessel distal and proximal to the pseudoaneurysm to prevent its collateral filling [4]. Yet, surgical resection of the pseudoaneurysm and ligation of the CA stump or right hepatic artery (RHA) would be an alternate treatment option if TAE fails.

Up to the authors’ knowledge, we identified 46 cases of porta hepatis pseudoaneurysm (Table 1) [2,4,6-24]. A total of 52.1% (24 cases) were males, age range from 39 to 82 years, with a mean age of 56.3 years, and 47.9% (21 cases) were females, age range from 37 to 88 years and a mean age of 61.1 years. The most commonly involved vessel was RHA (37 cases, 80.4%), followed by CA (eight cases, 17.4%), while left hepatic artery involvement was reported in one case. Forty-one cases were managed through the laparoscopic approach while five cases were done through open resection and the time of presentation ranged from day 1 up to 26 months post-procedure (mean of 60.4 days). The most common documented presentation was haemobilia, as reported in 31 cases (78.3%). Furthermore, abdominal pain, sepsis, jaundice, and hypotension were encountered, with only one case in the last five years presenting with hematochezia. In 12 cases, iatrogenic bile duct injury was considered, however, no case documented the presence of bile collection/biloma. Two out of 46 cases (4.3%) experienced an increased pseudoaneurysm size; however, four cases confirmed recurrence of bleeding episodes (8.7%). Forty-one cases (89.1%) were successfully managed by TAE, while three cases (6.5%) required surgical ligation, two cases (4.3%) were treated by percutaneous direct puncture and one case (0.1%) was managed conservatively. A favorable outcome was achieved in 97.8% of reported cases.

**Table 1:** A literature review of pseudoaneurysms of cystic artery or hepatic arteries following cholecystectomy CA: cystic artery; RHA: right hepatic artery; LHA: left hepatic artery; TAE: trans-arterial embolization; NBCA: n-butyl-2-cyanoacrylate; PDP: percutaneous direct puncture; LC: laparoscopic cholecystectomy; OC: open cholecystectomy; M: male; F: female; m: mean; RUQ: right upper quadrant pain

Author/year of the article	Number of cases	Age/gender	Type of surgery	Time to presentation	Presenting complaint	Bile duct Iatrogenic injury	Vessel involved	Increased aneurysms' size	Treatment
Rossini M et al., 2019 [2]	1	66/M	OC	28 Days	RUQ pain, haemobilia	No	CA	No	TAE/Coils
Machado NO et al., 2017 [4]	1	70/F	LC	14 Days	Abdominal pain	No	RHA	No	TAE/Coils
Gachabayov M et al., 2017 [12]	1	57/M	LC	15 Days	Haemobilia, jaundice, abdominal pain	No	RHA	Yes	TAE/Alcohol particles + Surgical ligation
Wen F et al., 2016 [23]	14	49 m/10 F, 4M	LC	21 Days (m)	Haemobilia, abdominal pain, jaundice	No	RHA	No	TAE/Coils
CreTu OM et al., 2017 [11]	1	55	LC	22 Months	Haemobilia + upper abdominal pain	No	RHA	No	Surgical ligation
Ion D et al., 2016 [14]	1	58/M	OC	27 Days	Haemobilia + anemia + hematochezia	No	RHA	No	TAE
To K et al., 2018 [20]	1	56/M	LC	28 Days	Haemobilia + RUQ pain	No	CA	No	TAE/Coils + stent
Abiko T et al., 2020 [6]	1	60/M	LC	3 Days	Haemobilia + RUQ pain	No	CA	No	TAE
Choudhary A et al., 2017 [10]	1	42/F	LC	26 Months	RUQ pain + haematemesis	No	CA	No	TAE/coil + stent/distal bulge
Villa-Gomez G et al., 2018 [22]	1	39/M	LC	35 Days	Haematemesis + cholangitis	Yes	RHA	No	TAE
Badillo R et al., 2017 [9]	1	79/M	LC	15 Months	Haemobilia	No	CA	No	PDP
Arata R et al., 2020 [8]	1	88/F	LC	12 Days	Haemobilia +Abdominal pain + Back pain	No	CA	No	Conservative
Traa AC et al., 2020 [21]	1	76/F	LC	1 Day	Pain + haemobilia	No	RHA	No	TAE/Coil + stent/coil
Tiwari A et al., 2017 [19]	1	80/M	LC	30 Days	UGIB + hematochezia + shock	No	LHA	No	TAE
Yagihashi K et al., 2017 [24]	1	55/M	OC	7 Days	Fever + abdominal pain	No	RHA	No	PDP
Rege SA et al., 2017 [17]	1	40/F	LC	55 Days	Haematemesis + RUQ pain	No	CA	No	TAE/Coils
Alrajraji M et al., 2016 [7]	1	41/F	LC	8 Months	Haematemesis + melena (haemobilia)	No	RHA	Yes	Surgical ligation
Gandhi RJ et al., 2020 [13]	13	52m/3F, 10M	LC	9-30 Days (15.6days=m)	3 haematemesis, 2 melena, 4 sepsis + anemia, 4 hypotension	Yes (9 cases)	RHA	N/A	TAE (3 Coils,4 NBCA,6 Coil+NBCA)
Kassem TW, 2017 [15]	1	42F	LC	6 Months	Haematemesis	No	RHA	No	TAE/Coils
Rosa C et al., 2018 [18]	1	52F	OC	4 Months	Haematemesis + melena (haemobilia)	Yes	RHA	No	TAE/NBCA
Mahfooz F et al., 2020 [16]	1	82M	OC	2 Months	Abdominal pain + melena	Yes	CA	No	TAE

In this case, we are discussing an unusual presentation of CASP bleed, in which, two assumed pathological risk factors for CA/right hepatic artery pseudoaneurysm formation are present, namely, vascular injury by surgical clips and post-LC bile collection. In the related literature review, a similar presentation of fresh bleeding per rectum secondary to CASP was identified (Table 1).

## Conclusions

CASP is a rare complication of post-LC, yet potentially life-threatening, with possible delayed complications occurring months to years after the surgery. CASP is commonly present with gastrointestinal bleeding, upper abdominal pain, and jaundice. CASP is diagnosed by contrast CT and angiogram and treated accordingly. Clinicians and radiologists should be aware of this important entity and its variable manifestations to facilitate early treatment.
